# SCARA5 inhibits gastric cancer progression via epithelial-mesenchymal transition suppression

**DOI:** 10.7150/jca.52426

**Published:** 2021-03-01

**Authors:** Hangyu Zhang, Changgang Liu, Xinbo Wang, Yongfang Wang, Jie Zheng

**Affiliations:** 1 Department of Diagnostic Pathology, School of Basic Medical Sciences, Weifang Medical University, Shandong Province, China.; 2 Department of Surgical Oncology, Weifang People's Hospital, Weifang Medical University, Shandong Province, China.; 3 Key Lab of Neurological Disease and Regeneration & Repair, Weifang Medical University, Shandong Province, China.

**Keywords:** Gastric cancer, SCARA5, proliferation, migration and invasion, EMT

## Abstract

Scavenger receptor class A member 5 (SCARA5) has been reported to be implicated in several types of cancer. However, its biological roles and mechanism of SCARA5 in gastric cancer (GC) have not been elucidated. In the present study, SCARA5 expression was found to be downregulated in GC which was associated with promoter methylation. The protein level of SCARA5 was negatively associated with aggressive clinicopathological characteristics, as well as poor prognosis. Moreover, SCARA5 overexpression markedly suppressed the growth, migration and invasion of GC cell lines *in vitro*. Furthermore, upregulation of SCARA5 inhibited gastric tumor growth and metastasis in a xenograft model. Mechanistic analysis revealed that SCARA5 suppressed the migration and invasion of GC cells via inhibiting epithelial-mesenchymal transition (EMT) and inactivating MMP-2 and MMP-9. Taken together, these results demonstrated that SCARA5 might play vital roles in the GC genesis and progression and could serve as a potential biomarker for diagnosis and therapeutic target of GC.

## Introduction

Gastric cancer (GC) is one of most frequent malignant tumors of digestive tract. About 1,033,701 new GC cases and 782,685 deaths occur in 2018 worldwide 1. Since the early symptoms of GC are not obvious, most of GC patients are diagnosed at an advanced stage. In spite of significant progression in the therapy of GC, including surgical resection and chemotherapy, survival rate remains unsatisfactory mainly because of late detection and chemoresistance. Thus, better understanding the underlying molecular mechanisms of GC is necessary to identify new biomarkers for early diagnosis and novel target genes for treatment.

Scavenger receptors (SRs) are extracellular glycoproteins that are involved in the recognition and endocytosis of biomolecules, such as polysaccharides, lipids, polyribonucleotide, modified lipoproteins 2. Several discrete classes of the SRs have been identified based on their ability to recognize different forms of modified low density lipoprotein, including SCARA1 (SR-AII/II), SCARA2 (MARCO), SCARA3 (CSR1), SCARA4 (SRCL) and so on 3,4.

Scavenger receptor class A member 5 (SCARA5) was found to be a new member of the SRs in 2006 4. SCARA5 has been reported to function as a tumor suppressor gene in several types of cancer previously, such as human hepatocellular carcinoma (HCC) 5, colorectal cancer 6, glioma 7, lung cancer 7,8 and breast cancer^9^. The aberrant expression of SCARA5 has been identified in gastric cancer by gene microarray, SCARA5 was indicated to have potential tumor suppressive function in GC as it showed downregulated expression in tumors tissues 10. Gu et al found that the SCARA5 expression was among top 15 down-regulated differential expression mRNAs in stomach adenocarcinoma compared with paired non-tumor tissues through high-throughput RNA-sequencing 11. However, the clinical significance of SCARA5 in GC and its association with patient's prognosis remains unclear, the potential functions of SCARA5 in the development of GC and its underlying molecular mechanisms are poorly understood.

In the present study, we investigated the SCARA5 expression in GC tissues and cells and examined the correlation between SCARA5 expression and clinicopathological parameters. We also analyzed the effect of SCARA5 on cell proliferation, migration and invasion of GC. Moreover, the potential mechanism involved in SCARA5-mediated suppression of GC progression was also examined.

## Materials and Methods

### Patients and GC tissues

The inclusion criteria of patients were (1) pathological diagnosis of gastric adenocarcinoma. (2) Patients with adequate pathological tissue. (3) Patients signed informed consent. (4) Complete clinical data are available. (5) None of the patients received chemotherapy, radiotherapy or immunotherapy. These patients were excluded who did not sign the informed consent or with no enough tumor tissue samples. 36 fresh gastric adenocarcinoma and 16 nontumor tissue samples were obtained from Weifang People's Hospital and Affiliated Hospital of Weifang Medical University. The clinical parameters of these patients is described in detail in Supplementary Table S1. For immunohistochemistry (IHC) 81 cases of paraffin-embedded GC tissues and their corresponding nontumor gastric mucosa were collected from Affiliated Hospital of Weifang Medical University between 2016 and 2017. The study was approved by the Ethics Committee of Weifang Medical University.

### Exploring Cancer Genomics Data using Cbioportal

The cBioPortal for cancer genomics (http://www.cbioportal.org/) provides analysis and download of large cancer genomics datasets. In this study, we used cBioPortal to explore the genetic variation of SCARA5 in gastric cancer.

### Bioinformatic Analysis of Clinical Data

Gastric cancer dataset was obtained from The Cancer Genome Atlas (TCGA). The SCARA5 expression and association were examined by Gene Expression Profiling Interactive Analysis (GEPIA) (http://gepia.cancer-pku.cn/)^12^ and UALCAN dataset (http://ualcan.path.uab.edu/). Overall survival of GC patients was detected by Kaplan-Meier Plotter (http://kmplot. com/analysis/)^12^. The GEO dataset included GSE14210, GSE15459, GSE22377, GSE29272, GSE51105.

### Immunohistochemistry (IHC)

Paraffin-embedded tissue sections were immunostained with SCARA5 polyclonal antibodies (1:200, Abcam, London, UK) and Ki-67(working fluid, maxim, fujian, China). All staining were performed according to the manufacturer's protocol. The results were evaluated and graded for staining intensity by two experienced pathologists. The staining intensity was graded into 0 (negative staining), 1 (weak staining), 2 (moderate staining), and 3 (strong staining). Percentage score was assigned as 1 (<25%); 2 (25-50%); 3 (51-75%); and 4 (76-100%). A final score was achieved by multiplying the scores for intensity and percentage. The SCARA5 expression level was considered low or none when the final scores were < 4 and high when the final scores were ≥4.

### RNA extraction and quantitative reverse transcription PCR (qRT-PCR)

Total RNA was isolated using Trizol reagent (Invitrogen, Carlsbad, California, US). Then, cDNA was synthesized from 1 μg total RNA with ReverTra Ace qPCR RT Kit (Toyobo, Osaka, Japan). QRT-PCR was performed using a FastStart Universal SYBR Green Master Kit (Roche, Mannheim, Germany) with a CFX96^TM^ system (Bio-Rad).

The relative SCARA5 expression was calculated by the 2^-ΔΔCT^ method. The primers for PCR were as follows: SCARA5 forward: 5´-CTGTCCAAGCTGAACCTGTGT-3´; reverse: 5´-AGATGAAGATGCCCACAAGAAT-3´; GAPDH forward: 5´-GCACCGTCAAGGCTGAGAAC-3´, reverse: 5´- TGGTGAAGACGCCAGTGGA-3´.

### DNA isolation, bisulfite modification of DNA, methylation-specific PCR (MSP)

Genome DNA was extracted from all specimens using the QIAamp DNA Mini kit (Qiagen, Germantown, MD, USA) according to the protocols. Bisulfite conversion of the genomic DNA was performed using EZ DNA Methylation-Gold Kit (Zymo Research, Orange, CA, USA).

MSP was performed using the following primers: methylated SCARA5 forward: 5´-GTTTAGCGGGCGTTTTTATACG-3´, reverse: 5´-AACAAAACCACAAAAACCACTTC-3´; unmethylated SCARA5 forward: 5´- AGTGGGTGTTTTTATATGGGGTT-3´, reverse: 5´- ACTCCCTATCCTTAATACCTAATCCT-3´.

### Cell culture and transfection

Human immortalized gastric cells (GES) and SGC7901, MKN45 and BGC823 GC cell lines were purchased from the American Type Culture Collection (ATCC, Manassas, VA, USA) and Shanghai Cancer Institute (Shanghai, China). The full-length SCARA5 coding sequences were cloned into the pcDNA3.1 (+) plasmid (pcDNA3.1 (+)-SCARA5), and the empty plasmid pcDNA3.1 (+) was used as a negative control. The transfection was performed using lipofectamine 2000 (Invitrogen) accordance to the manufacturer's instructions.

### 5-aza-2'-deoxycytidine (Aza) treatment

The BGC823, MKN45and SGC7901 cells were treated with 10 µM DNA methyltransferase inhibitor Aza, (Sigma-Aldrich, St. Louis, MO, USA) for 72h, then genomic and DNA total RNA were extracted. The effect of Azaon the methylation status and the expression of SCARA5 gene was analyzed by MSP and qRT-PCR in GC cells.

### Cell proliferation assays

Cell proliferation was analyzed using Cell-Light EdU Apollo 488 *In vitro* Kit (RiboBio, guangzhou, China) and Cell Counting Kit-8 (CCK-8; Dojindo, Gaithersburg, MD, USA) in accordance with the manufacturer's instructions.

### Cell Migration and Invasion Assays

Transwell inserts (8.0 µm, Corning, NY, USA) was used to perform migration assays, and the inserts coated with a Matrigel matrix (BD Science, Sparks, MD, USA) were used for invasion assay. Cell migration and invasion assays were performed as described previously 13.

### Enzyme-linked immunosorbent assay (ELISA)

The supernatants of cell culture were collected 48h after transfection and the concentrations of MMP-2 and MMP-9 in the conditioned media were determined by ELISA kits (R&D Systems, Minneapolis, USA) according to the manufacturer's instructions.

### *In vivo* tumorigenic and metastasis assay

BGC823 cells were transfected with a lentiviral SCARA5 overexpression vector (LV-SCARA5) or empty vector (LV-NC; GenePharma, Shanghai, China), and then these cells were injected into the right dorsal flanks (n=6 each) or the lateral tail vein (n=6 each) of 4-week-old male Nu/Nu mice (Vital River, Beijing, China). Tumor growth was measured every week and tumor volumes (V) were calculated as V = (tumor length × width^2^)/2. Six weeks after injection, we sacrificed the mice and harvested the tumor nodules and lungs which were stained by Hematoxylin and Eosin (HE) for histological evaluation. Tumor nodules and metastatic loci were confirmed and the number of metastatic loci was detected under microscope. All animal procedures were approved by the Animal Care and Use Committee of Weifang Medical University.

### Western Blot

Total protein was extracted from cells or tissues and were resolved by electrophoresis for immunoblotting with antibodies against SCARA5 (1:1000, Abcam), E-cadherin and GAPDH (1:1000, Proteintech, Wuhan, China), N-cadherin, Vimentin, Snail, Twist, Zeb1, MMP-2and MMP-9 (1:1000, Cell Signaling Technology, Danvers, MA, USA).

### Statistical Analysis

GraphPad Prism 5 software (San Diego, CA, USA) was used for statistical analysis. Analysis of the differences between the two groups was determined by Student's t-test. Chi-square and Fisher's exact test were used to analyze the association between SCARA5 expression and the clinicopathologic parameters. The patients' survival was analyzed by the Kaplan-Meier method and log-rank test. *P*<0.05 was considered statistically significant.

## Results

### Decreased expression of SCARA5 in gastric cancer tissues and cells

**Figure 1:**
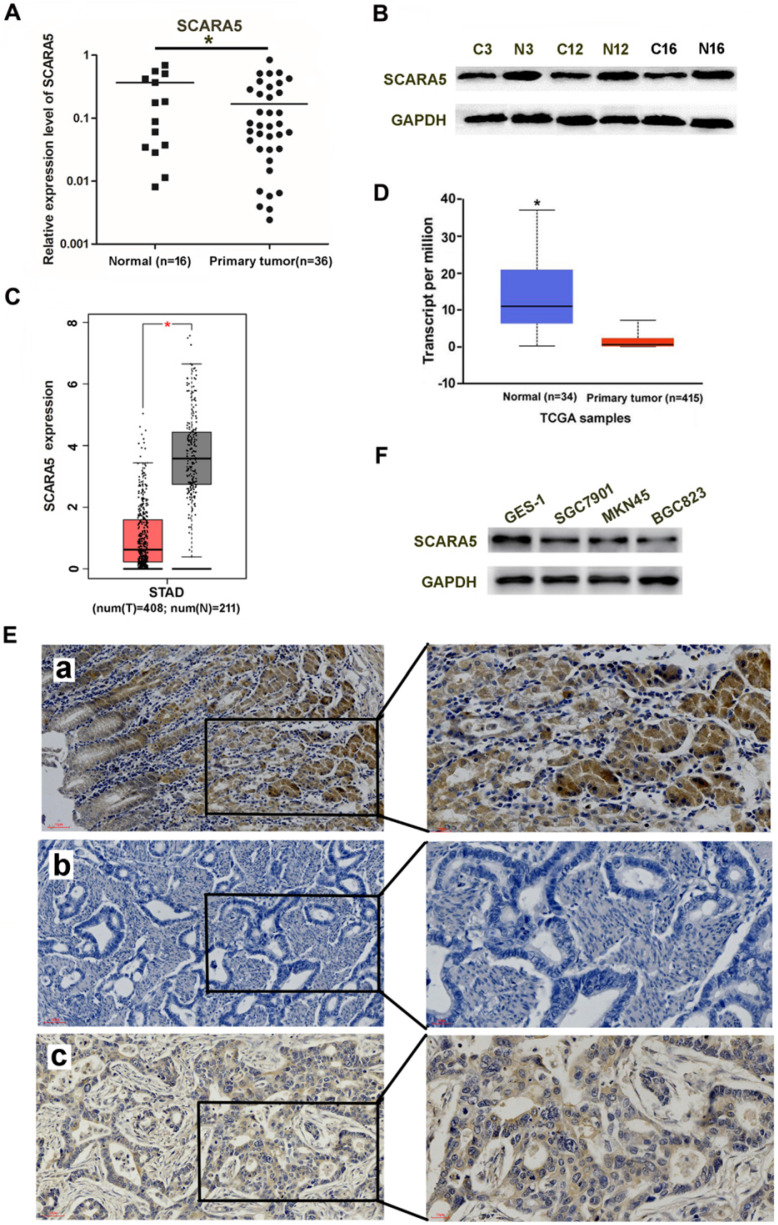
** SCARA5 expression was downregulated in gastric cancer tissues.** A, B, SCARA5 expression level in gastric cancer tissues and adjacent normal mucosa was detected by qRT-PCR (A) and western blot (B). C,D, The average SCARA5 expression from gastric cancer tissues and adjacent normal tissues was examined by GEPIA (C) and UALCAN dataset (D). E, Representative IHC images of high or low SCARA5 expression in 81 pairs of GC tissues and adjacent normal mucosa. The *scale bar* represents 50 µm and 20 µm respectively. a positive staining of SCARA5 in adjacent normal tissues; b negative staining of SCARA5 in gastric cancer tissues; c positive staining of SCARA5 in gastric cancer tissue. F, Western blot analysis of SCARA5 protein expression in a panel of gastric cell lines. GAPDH was used as an internal control. Three independent experiments were performed and the data are represented as mean± SD, all *P<0.05.

The mRNA and protein level of SCARA5 in gastric cancer tissues (n=36) and adjacent non-tumor tissues (n=16) was detected by qRT-PCR and western blot. Results demonstrated that the SCARA5 expression was significantly lower in gastric cancer tissues than in the adjacent non-tumor tissues** (Figure 1A, B)**. SCARA5 expression was further analyzed in GC tissues and human normal stomach tissues from TCGA dataset. The SCARA5 expression was significantly downregulated in GC specimens compared with normal tissues** (Figure 1C, D)**. The expression of SCARA5 was also examined by IHC analysis. The percentage of positive tumor cells and the staining intensity for each sample were recorded. The low or none expression rate of the SCARA5 was 60.5% (49/81) in GC cases and 34.6% (28/81) in adjacent non-tumor tissues **(Figure 1E)**.

To further demonstrate the function of SCARA5 in GC, western blot was also performed to measure SCARA5 expression in three GC cell lines and the human immortalized gastric cells line GES-1. The results found that the GC cell lines have lower expression of SCARA5 than GES-1**(Figure 1F)**. These results demonstrated notable downregulation of SCARA5 in both primary GC cancer tissues and human GC cell lines.

### DNA methylation contributes to the decreased expression of SCARA5 in GC

**Figure 2:**
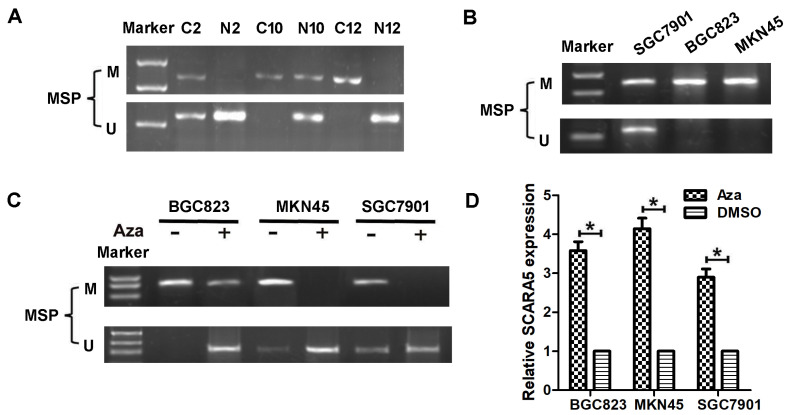
** DNA methylation regulates SCARA5 expression in GC.** A, MSP analyses of SCARA5 promoter methylation in gastric cancer tissues and adjacent normal mucosa. C, gastric cancer tissues; N, adjacent normal mucosa. B, MSP analyses of SCARA5 promoter methylation in GC cell lines. C, MSP indicate demethylation by Aza restored SCARA5 expression in GC cell lines. The SCARA5 expression was assessed by qRT‐PCR analysis with or without Aza treatment in GC cell lines. Error bars represent the SD from three independent experiments. All *P<0.05.

Previous reports have found that SCARA5 is downregulated in several types of malignant tumors owing to SCARA5 promoter methylation, to investigate whether the decreased SCARA5 expression is correlated with promoter hypermethylation, DNA samples from 36 GC and 16 non-cancerous gastric mucosa tissues were subjected to MSP assay to detect the methylation frequency of CpG island within SCARA5 gene promoter. Methylation was detected in 20 (55.6%) GC tissues, while only in 2 (12.5%) non-cancerous gastric mucosa **(Figure 2A)**.

The methylation status of SCARA5 promoter with MSP in BGC823, MKN45 and SGC7901 cell lines. The promoter methylation correlated was found in these GC cell lines **(Figure 2B)**. Moreover, these GC cells were treated with Aza which resulted in the decreased SCARA5 methylation** (Figure 2C)** and SCARA5 expression was restored or strongly increased **(Figure 2D)**. In conclusion, these results demonstrate that SCARA5 is a potential tumor suppressor that is inhibited by promoter hypermethylation in GC.

### The correlation of SCARA5 expression with clinicopathological parameters and prognosis

We next detected the association of SCARA5 expression with the clinicopathological parameters of GC patients. Statistical analysis found that the decreased expression of SCARA5 was correlated with tumor size (*P* = 0.0396), lymph node metastasis (*P* = 0.0254) and higher TNM stage (*P* = 0.0366), but not with patients' age, gender, Lauren's Classification, or differentiation status** (Table 1)**.

**Figure 3:**
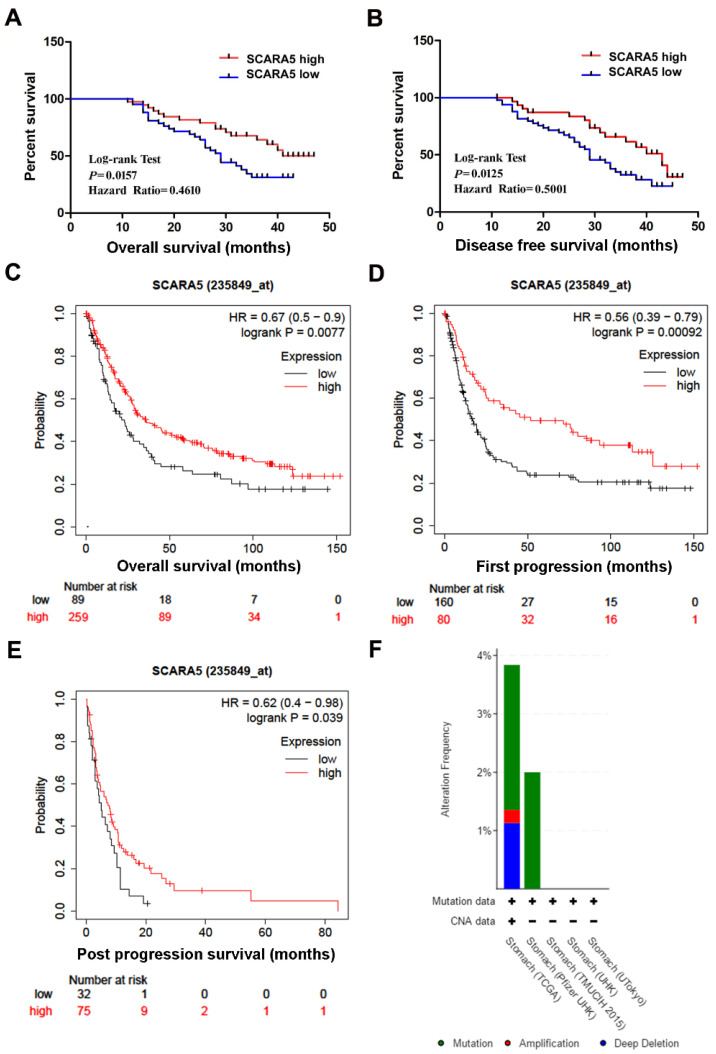
** Down-regulation of SCARA5 is correlated with poor prognosis of GC patients.** A, B, Kaplan‐Meier curves with univariate analysis of overall survival and disease‐free survival based on the SCARA5 expression. C, D, E, Low SCARA5 expression is correlated with poor overall survival rate (C), first progression rate (D) and post progression survival rate (E) in GC patients. Prognosis data was analyzed using the Kaplan-Meier Plotter database. F, Genetic alterations detection of SCARA5 Gene in gastric cancer using cBioPortal.

Furthermore, Kaplan-Meier survival analysis and log-rank test found that patients with low SCARA5 expression had poorer overall survival (Hazard Ratio=0.4610, *P* = 0.0157,** Figure 3A**) and disease-free survival (Hazard Ratio=0.5001, *P* = 0.0125, **Figure 3B**). Moreover, the overall survival (OS), the first progression(FP) and the post progression survival (PPS) in SCARA5 low patients were much lower than in SCARA5 high patients analyzed by Kaplan-Meier Plotter** (Figure 3C-E)**. These results suggested that SCARA5 could be a potential biomarker to predict the prognosis of GC patients.

### Genetic Alterations of SCARA5 Gene in GC

CBioPortal was used to investigate the genetic changes of SCARA5 gene in 5 GC studies. In 708 cases of gastric cancer, the genetic change rate of SCARA5 ranged from 0 to 3.84%, with an average of 2.68%. Of 19 cases of genetically altered gastric cancer, 13 cases had SCARA5 mutation, 5 cases had deep deletion and 1 case had amplification **(Figure 3F)**.

### SCARA5 inhibits GC cell growth *in vitro* and* in vivo*

**Figure 4:**
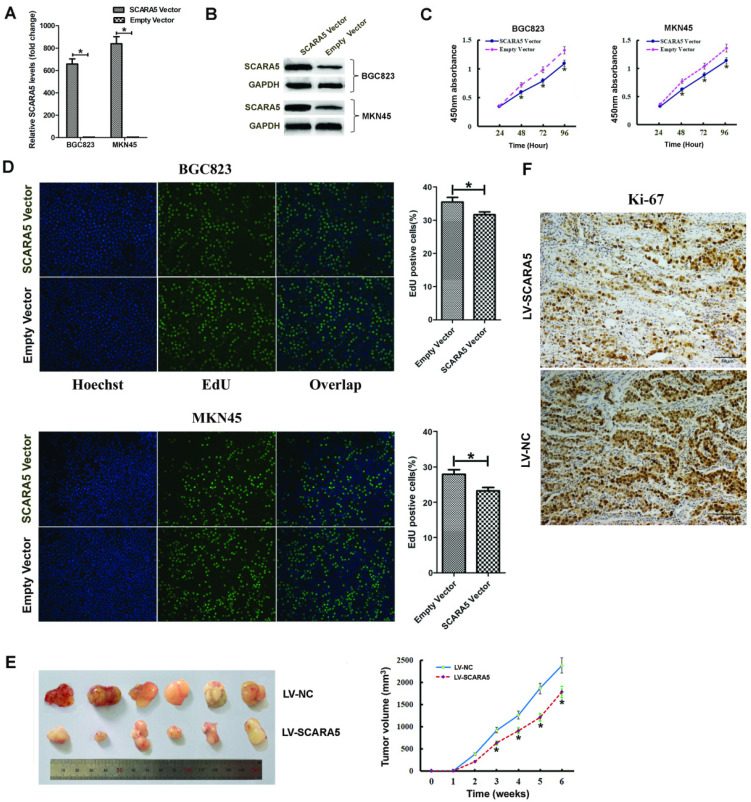
** Effect of SCARA5 on proliferation of GC cells *in vitro* and *in vivo*.** A, B, The BGC823 and MKN45 cells were transfected with SCARA5‐overexpressing plasmid and empty plasmid, SCARA5 expression was examined by qRT‐PCR (A) and Western blot (B).C, D, Cell viability was determined by CCK-8 (C) and EdU (D) assay and the data demonstrated that overexpression of SCARA5 could suppressed cell the growth of BGC823 and MKN45 cells. E, Up-regulated expression of SCARA5 in BGC823 cells attenuated tumorigenesis *in vivo*. Tumor volumes were measured every one week. F, Ki-67immunohistochemistry examination demonstrated that tumor cells in the SCARA5-overexpressing group revealed a lower positivity rate than those in the negative control group (magnification ×200). Three independent experiments were performed and the data are represented as mean± SD. All *P<0.05.

In order to investigate the biological functions of SCARA5 in the proliferation of GC cells, we transfected GC cells with SCARA5-overexpressing plasmid or empty plasmid. The overexpression efficiency of SCARA5 was determined 48h by qRT-PCR and western blot after transfection** (Figure 4A-B)**. EdU staining and CCK-8 assay were performed in BGC823 and MKN45 cells. CCK-8 assays demonstrated that SCARA5 overexpression inhibited GC cell proliferation **(Figure 4C)**. Consistent with the CCK-8 results, the EdU staining also found that SCARA5 suppressed GC cell proliferation **(Figure 4D)**.

To further examine the effect of increased SCARA5 expression on GC cell growth* in vivo*, the xenograft nude mouse model experiment was performed and the subcutaneous tumor nodules derived from the LV-SCARA5 group grew slower than those derived from the LV-NC group **(Figure 4E)**. Meanwhile, the Ki-67 expression was lower in SCARA5-overexpressing cells from tumor nodules than those cells of negative control group **(Figure 4F)**.

### SCARA5 suppresses the invasion and metastasis ability of GC cells *in vitro* and* in vivo*

**Figure 5:**
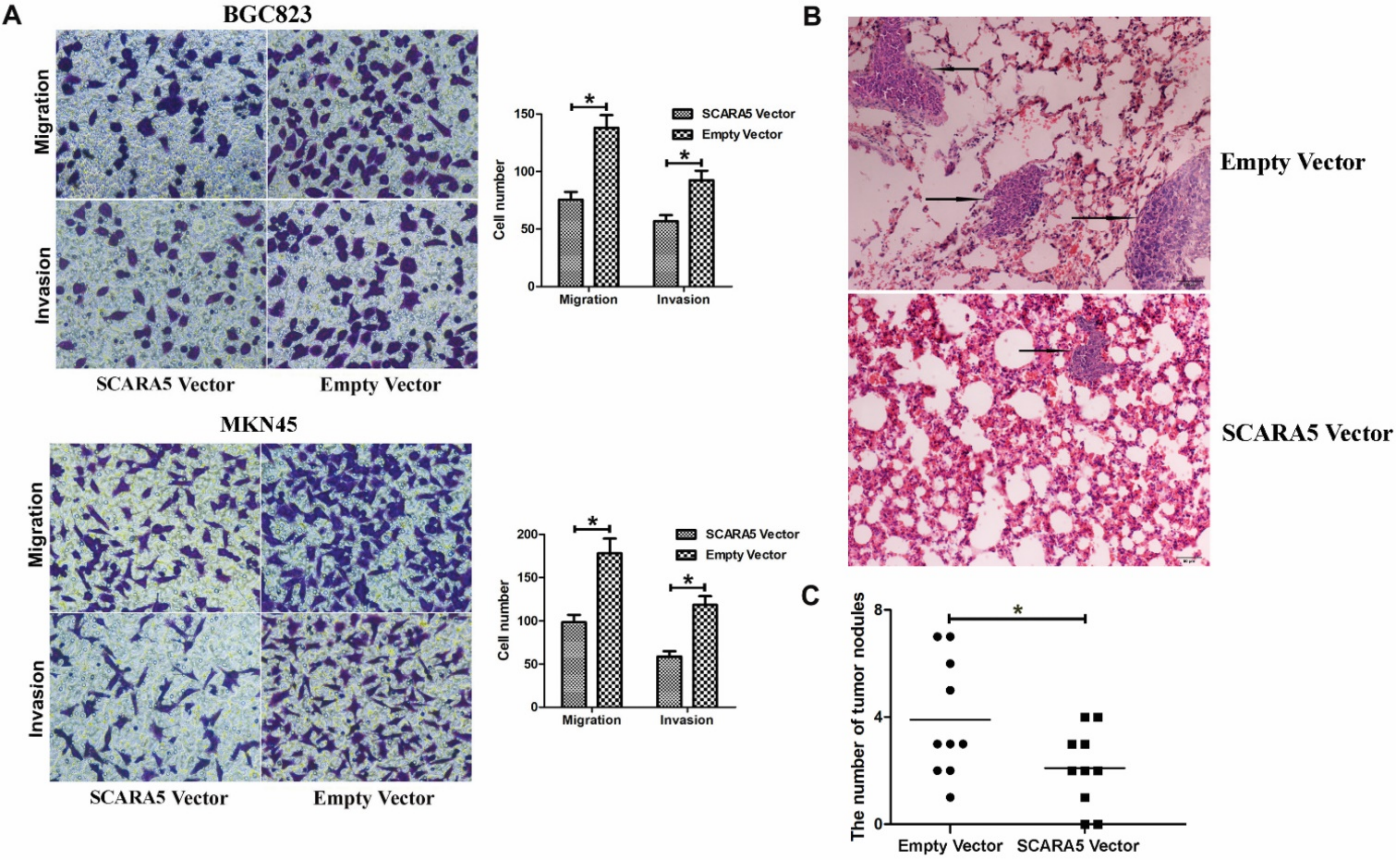
** SCARA5 inhibited the invasion and metastatic ability of gastric cancer cells* in vitro* and *in vivo*.** A, The migration and invasion ability of BGC823 and MKN45 cells was significantly suppressed after SCARA5 was overexpressed detected by the transwell assay. B, Representative HE staining of lungs separated from the Nu/Nu mice. The metastatic foci (the arrow) of lung in the LV-SCARA5 group were less and smaller than those in the LV-NC group (HE magnification ×200). C, Numbers of lung metastatic foci of the two groups were calculated. Error bars represent the SD from three independent experiments. All *P<0.05.

Considering that the inverse association between SCARA5 expression level and metastasis, we detected the function of SCARA5 in the migration and invasion of GC cells. The results found that overexpression of SCARA5 significantly promoted the migration and invasion abilities of BGC823 and MKN45 cells** (Figure 5A)**.

Next, we explored potential effects of SCARA5 on cells' metastasis *in vivo*, BGC823 cells transfected with LV-SCARA5 or LV-NC were injected to the tail vein of the nude mice. Hematoxylin and eosin (HE) staining demonstrated that less and smaller volumes metastatic loci were found in the lungs of the mice in the LV-SCARA5 group than those in the LV-NC group **(Figure 5B,C)**. These results showed that SCARA5 played dominant roles in suppressing GC progression via attenuating the invasion and metastasis abilities of GC cells.

### SCARA5 inhibited GC aggressiveness via EMT

**Figure 6:**
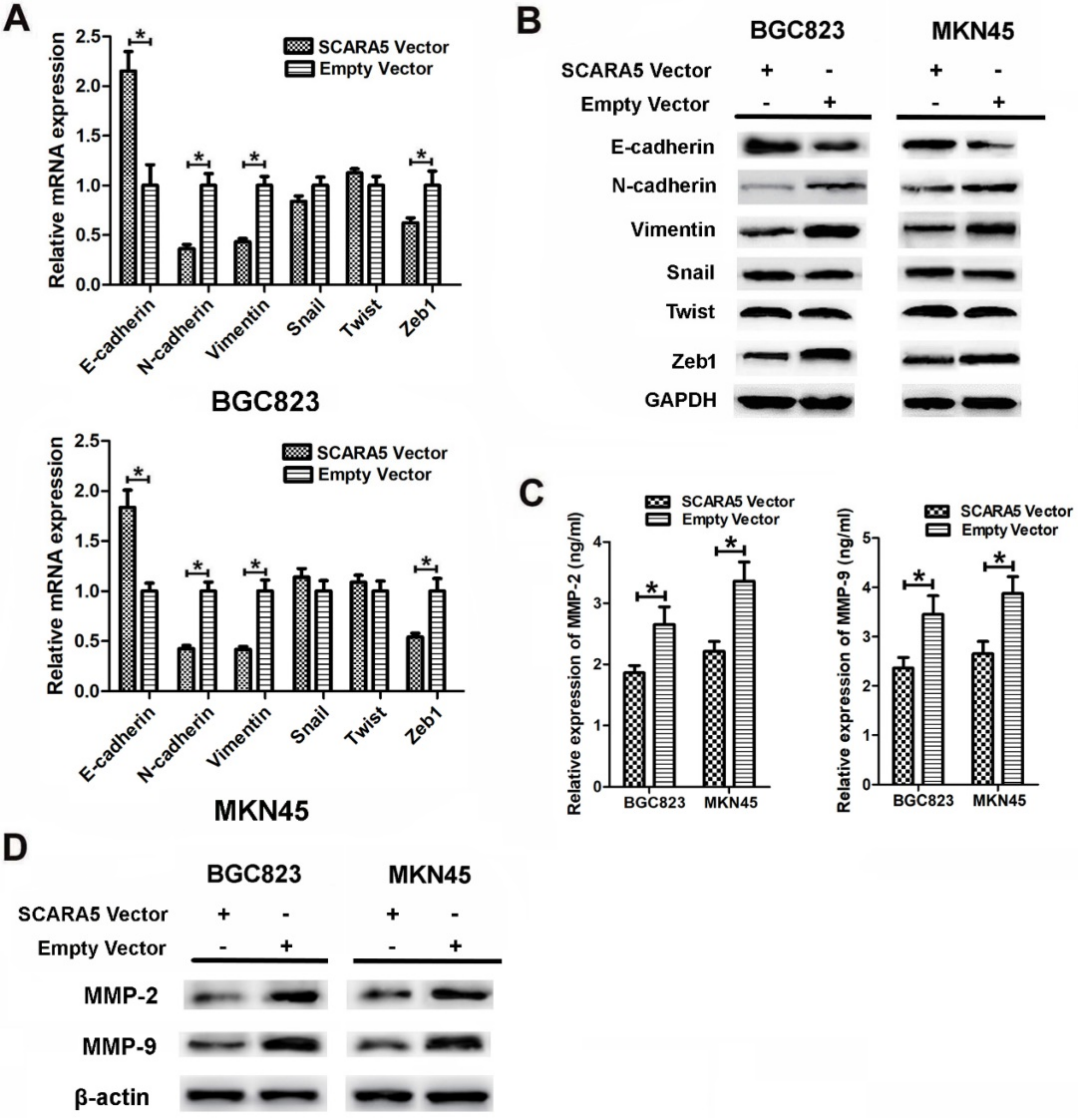
Overexpression of SCARA5 inhibited GC aggressiveness through EMT process and regulating the MMP-2 and MMP-9 expression. A, B, The expression of EMT-associated markers (E-cadherin, N-cadherin and Vimentin) and transcription factors (Snail, Twist and Zeb1) was assessed by qRT-PCR (A) and western blot (B). C, The expression of MMP-2 and MMP-9 was examined in the supernatants of cultured GC cells by ELISA assay. D, The expression of MMP-2 and MMP-9 was detected in the transfected GC cells by western blot. Data are presented as the mean ± SD of at least three independent experiments. All *P<0.05.

To investigate the possible mechanism responsible for the roles of SCARA5 on GC cell migration and invasion, we assessed the effects of SCARA5 in the epithelial-mesenchymal transition (EMT) process. We performed qRT-PCR and western blot to detect the key EMT biomarkers expression level after SCARA5 was overexpressed in GC cells. QRT-PCR results found that, along with the increase of SCARA5, the expression of the epithelial marker E-cadherin increased significantly, while the mesenchymal markers vimentin and N-cadherin and transcriptional factor Zeb1 decreased dramatically **(Figure 6A)**, similar results were obtained by western blot analysis **(Figure 6B)**. Thus, SCARA5 plays an important role in the suppression of EMT, and SCARA5 might inhibit gastric cancer cells aggressiveness by influencing EMT initiation.

### SCARA5 is relevant to decreased activation of MMP-2 and MMP-9

Our results have demonstrated that overexpression of SCARA5 suppresses the invasive ability of GC cells and MMPs are proved to be involved in GC invasion. The expression of MMP-2 and MMP-9 was assessed in the supernatants of cultured BGC823 and MKN45 cells after transfection by ELISA assay. The MMP-2 and MMP-9 expression were downregulated in SCARA5 overexpression cells** (Figure 6C)**. Furthermore, western blot results also demonstrated that SCARA5 upregulation reduced the expression of MMP-2 and MMP-9 protein** (Figure 6D)**. Therefore, MMP-2 and MMP-9 are vital factors in SCARA5 that inhibits the invasiveness of GC cells.

## Discussion

SCARA5 was downregulated and acted as a tumor suppressor gene in many kinds of cancers. Huang et al found that SCARA5 suppressed human hepatocellular carcinoma (HCC) cell growth, invasiveness and lung metastasis *in vitro* and *in vivo* 5. SCARA5 significantly inhibited cell proliferation, invasion, and migration, and induced G0/G1 arrest and apoptosis of breast cancer cells 9. SCARA5 has been observed to be down-regulated expression in GC compared with adjacent non-tumor tissues through microarray 10 and high-throughput RNA-sequencing 11. Huang *et al* found SCARA5 to be markedly downregulated in 62 (76.5%) of the 81 matched GC tumor tissues by IHC staining 5. However, the expression, biological function and clinical significance of SCARA5 in the progression and metastasis of gastric cancer have not been fully understood.

In this study, for the first time, we demonstrated that SCARA5 played a tumor suppressor role in GC.We identified that the mRNA and protein levels of SCARA5 were both down-regulated in GC samples and cell lines, and it's consistent with the TCGA database. Promoter methylation accounts for the reduced SCARA5 expression in several cancers 5,14,15, we detected SCARA5 down-regulation correlated with promoter hypermethylation in GC cell lines and tissues. Demethylation treatment significantly restored SCARA5 expression, suggesting that promoter methylation could be the main mechanism underlying SCARA5 inactivation in GC.

Kaplan-Meier survival analysis of GC patients demonstrated that low expression of SCARA5 had significantly reduced survival rates. Tumor progression is a complicated process that encompasses cell growth, basement membrane degradation, cell migration, invasion and metastasis. The potential functions of SCARA5 in gastric cancer cell growth, migration and invasion were further examined by overexpression plasmid transfection. Our data showed that upregulated expression of SCARA5 significantly suppressed proliferation, migration and invasion of gastric cancer cells *in vitro*. Moreover, stable overexpression of SCARA5 reduced inhibited tumor growth and suppressed tumor metastasis to the lungs* in vivo*. These results provide strong evidence that SCARA5 is correlated with GC initiation and progression.

The mechanisms of SCARA5 as a tumor suppressor are not fully understood. The epithelial-mesenchymal transition (EMT) is a prerequisite physiological process for metastasis in most cancer 16. EMT could induce epithelial cells to change to a mesenchymal phenotype which was conducive to tumor progression via decreasing cell adhesion and elevating cell invasive capacity 17. Emerging evidence has suggested the correlation of SCARA5 with EMT in malignant tumors. SCARA5 downregulation was essential for EMT-induced migration in A549 cells and SCARA5 was a direct Snail1 target modulating cancer cell mobility during EMT 8. SCARA5 also modulated the expression of epithelial-mesenchymal transition-related proteins in thyroid cancer 18. These results demonstrated that SCARA5 could inhibit cell invasion and metastasis by modulating the actin cytoskeleton in cancer cell lines. In the present research, we found that overexpression of SCARA5 significantly altered the mRNA and protein levels of the epithelial and mesenchymal markers and transcriptional factor Zeb1 in GC cells. These results provided powerful evidence that SCARA5 played an important role in the initiation of the EMT process, by which it might exert the function of suppressing GC cell invasion and metastasis.

It has been clearly established that cancer cell growth and invasion is subordinated to extracellular matrix and basal membrane degradation by matrix metalloproteinases (MMPs). MMP-2 and MMP-9 are members of the MMPs family and have been reported to be overexpressed in gastric cancer. Overexpression of SCARA5 downregulated MMP-2, MMP-3 and MMP-9 in breast cancer cell 9. In human hepatocellular carcinoma, SCARA5 knockdown markedly enhanced the MMP-9 activity, but not that of MMP-2 5. In this study, reduced MMP-2 and MMP-9 expression was found in SCARA5-upregulated group which indicated that MMP-2 and MMP-9-mediated degradation of the extracellular matrix was involved in the tumor suppressive function of SCARA5.

In conclusion, we identified that SCARA5 was a new tumor suppressor gene that was silenced by promotor hypermethylation and might participate in the tumorigenesis and progression of GC. Its downregulated-expression was correlated with larger tumor size, lymph node metastasis and higher TNM staging. Overexpression of SCARA5 in GC might inhibit invasion and metastasis by reducing the EMT, and inactivated MMP-2 and MMP-9. Taken together, we proposed that SCARA5 acted as a tumor suppressive factor, and it had great potential to be a gene target in cancer treatment and a clinical biomarker in diagnosing gastric cancer.

## Supplementary Material

Supplementary table S1.

## Figures and Tables

**Table 1:** Association between SCARA5 expression and clinicopathological characteristics in patients with GC

Characteristics	No. of cases	Expression of SCARA5		*P* value
		High (%)	Low or none (%)	
**Age(years)**				0.8194
≥60	46	19 (41.3)	27 (58.7)	
<60	35	13 (37.1)	22 (62.9)	
**Gender**				1.0000
Male	45	18 (40.0)	27 (60.0)	
Female	36	14 (38.9)	22 (61.1)	
**Tumor size(cm)**				**0.0396**
≥5	43	12 (27.9)	31 (72.1)	
<5	38	20 (25.6)	18 (74.4)	
**Lauren's Classification**				0.4950
Intestinal	45	16 (35.6)	29 (64.4)	
Diffuse	36	16 (44.4)	20 (55.6)	
**Differentiation status**				0.6458
Well/moderate	34	12 (35.3)	22 (64.7)	
Poor	47	20 (42.6)	27 (57.4)	
**Lymph node metastasis**				**0.0254**
Negative	43	22 (51.2)	21 (48.8)	
Positive	38	10 (26.3)	28 (73.7)	
**TNM stage**				**0.0366**
I+II	33	18 (54.5)	15 (45.5)	
III+IV	48	14 (36.8)	34 (63.2)	

## Abbreviations

SCARA5: Scavenger receptor class A member 5
mRNA: messenger RNA
IHC: immunohistochemistry
qRT-PCR: quantitative reverse transcription PCR
EMT: epithelial-mesenchymal transition
MMP: matrix metalloproteinase
ELISA: Enzyme-linked immunosorbent assay
HE: Hematoxylin and eosin
Aza: 5-aza-2´-deoxycytidine
