# Supplementary material for: SCARA5 inhibits gastric cancer progression via epithelial-mesenchymal transition suppression
Source: J Cancer. 2021 Mar 1;12(8):2412–21. doi: 10.7150/jca.52426 (PMC7974898; doi:10.7150/jca.52426)
Supplement: Supplementary file 1 — Supplementary table S1. [file jcav12p2412s1.pdf]

Supplementary Table S1 Clinicopathological characteristics of the patients with GC

Characteristics	No. of cases
Age(years)	
≥60	22
<60	14
Gender	
Male	26
Female	10
Tumor size(cm)	
≥5	20
<5	16
Lauren’s Classification	
Intestinal	15
Diffuse	21
Differentiation status	
Well/moderate	12
Poor	24
Lymph node metastasis	
Negative	8
Positive	28
TNM stage	
Ⅰ+Ⅱ	11
Ⅲ+Ⅳ	25
